# West African medicinal plants and their constituent compounds as treatments for viral infections, including SARS-CoV-2/COVID-19

**DOI:** 10.1007/s40199-022-00437-9

**Published:** 2022-04-27

**Authors:** Temidayo D. Popoola, Peter A. Segun, Edmund Ekuadzi, Rita A. Dickson, Olanrewaju R. Awotona, Lutfun Nahar, Satyajit D. Sarker, Amos A. Fatokun

**Affiliations:** 1 Centre for Natural Products Discovery (CNPD), School of Pharmacy and Biomolecular Sciences, Liverpool John Moores University, James Parsons Building, Byrom Street, Liverpool, L3 3AF UK; 2 Department of Pharmacognosy, Faculty of Pharmacy, Olabisi Onabanjo University, Ogun State, Sagamu Campus, Nigeria; 3 Department of Pharmacognosy, Faculty of Pharmacy and Pharmaceutical Sciences, Kwame Nkrumah University of Science and Technology, Kumasi, Ghana; 4 Department of Pharmacy and Pharmaceutical Sciences, College of Health Sciences, Legacy University, No. 55, Kairaba Avenue, Fajara, Banjul, The Gambia; 5 Laboratory of Growth Regulators, Institute of Experimental Botany, ASCR & Palacký University, Šlechtitelů 27, 78371 Olomouc, Czech Republic

**Keywords:** Antiviral, Medicinal Plants, Traditional Medicine, SARS-CoV-2, COVID-19, West Africa

## Abstract

**Objectives:**

The recent emergence of the COVID-19 pandemic (caused by SARS-CoV-2) and the experience of its unprecedented alarming toll on humanity have shone a fresh spotlight on the weakness of global preparedness for pandemics, significant health inequalities, and the fragility of healthcare systems in certain regions of the world. It is imperative to identify effective drug treatments for COVID-19. Therefore, the objective of this review is to present a unique and contextualised collection of antiviral natural plants or remedies from the West African sub-region as existing or potential treatments for viral infections, including COVID-19, with emphasis on their mechanisms of action.

**Evidence acquisition:**

Evidence was synthesised from the literature using appropriate keywords as search terms within scientific databases such as Scopus, PubMed, Web of Science and Google Scholar.

**Results:**

While some vaccines and small-molecule drugs are now available to combat COVID-19, access to these therapeutic entities in many countries is still quite limited. In addition, significant aspects of the symptomatology, pathophysiology and long-term prognosis of the infection yet remain unknown. The existing therapeutic armamentarium, therefore, requires significant expansion. There is evidence that natural products with antiviral effects have been used in successfully managing COVID-19 symptoms and could be developed as anti-COVID-19 agents which act through host- and virus-based molecular targets.

**Conclusion:**

Natural products could be successfully exploited for treating viral infections/diseases, including COVID-19. Strengthening natural products research capacity in developing countries is, therefore, a key strategy for reducing health inequalities, improving global health, and enhancing preparedness for future pandemics.

**Graphical abstract:**

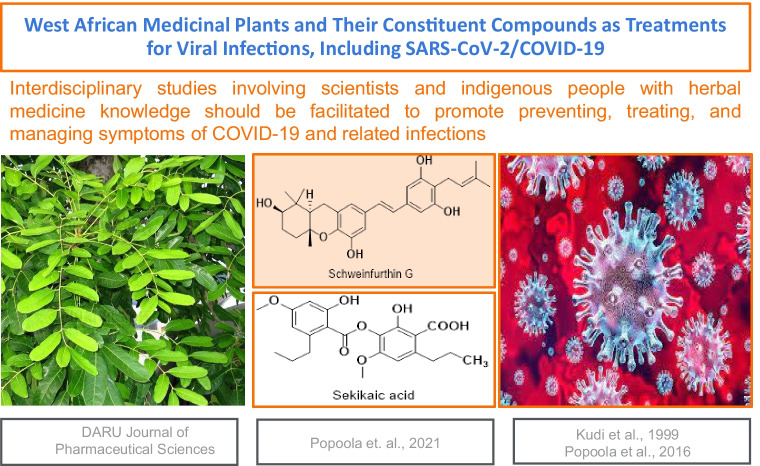

## Introduction: burden of SARS-CoV-2

The emergence in late 2019 of the novel SARS-CoV-2 virus (named COVID-19) and its consequent worldwide transmission has led to a significant burden on health care systems in almost every country on planet earth [1, 2]. COVID-19, the disease caused by the virus, exponentially expanded from the first reported case in Wuhan, China, on the 31^st^ of December, 2019 to 364,191,494 confirmed cases and 5,631,457 deaths reported by the World Health Organisation (WHO) as of 28th January, 2022 [3]. So far in the course of the pandemic there has been a worrying trend of an abatement followed by a resurgence, especially in countries that were originally considered to have done very well in managing the pandemic, with the resurgence (termed “second or third or new waves”) being linked to several factors, including the emergence of new variants of the virus, differences in the extent and effectiveness of countries’ lockdown, quarantine and other preventive measures, overwhelming of healthcare capacity for treating the infected, and the fact that there are several aspects of the new infection that not much is known about to date.

SARS-CoV-2 is one of seven strains of coronaviruses (CoVs) recorded to date [4]. It belongs to Beta-coronaviruses-type Human Coronaviruses, the same group as the Middle East Respiratory Syndrome Coronavirus (MERS-CoV) and Severe Acute Respiratory Syndrome Coronavirus (SARS-CoV). Data from the WHO indicate that SARS and MERS coronaviruses were the most destructive strains of CoVs until the current outbreak. MERS has a mortality rate of 36% and SARS 10% [5].

Even though reasonable progress has been made against COVID-19 in terms of finding effective preventive measures with the introduction of vaccines and treatment measures owing to the identification of some drugs or drug combinations, there is to date continuing profound burden of the infection on health care systems, as well as attendant disruptions to living and livelihoods occasioned by the varying quarantine, lockdown, and social distancing measures introduced by countries. Therefore, there is still significant pressure on the WHO, governments, academic institutions, pharmaceutical industries, charities, and related organisations to find curative treatments (drugs) to complement the current armamentarium. The growing emergence of SARS-CoV-2 variants of concern also suggests vaccines will have to keep being modified to retain acceptable levels of effectiveness. While some countries are currently doing well in their vaccine roll-out programmes, many countries still appear to be struggling in this regard and, unfortunately, their infection and death rates continue to soar.

While the current realities and future threats of COVID-19 are shared globally, the ominous potential for the developing countries to be disproportionately hard-hit in the short- and long-term presents a frightening spectre, although it currently appears the numbers of cases in those countries are generally lower than for other regions of the world. The higher vulnerability of developing countries is due to several factors. Chiefly, health systems in those countries are weak, fragile and lack the capacity [6] to contain full-blown infections within populations. These systems are already burdened by a slew of other infectious diseases such as malaria, tuberculosis, and Human Immunodeficiency Virus (HIV), amongst others [7]. Also, the poverty status and the culture in these countries [8] make extended lockdowns and social distancing measures near impossible. It is, therefore, important that solutions proposed for tackling SARS-CoV-2 and its effects in developing countries are sensitive to the dynamics of existing opportunities and challenges in those environments and how these might impact the effectiveness, affordability and accessibility of therapeutic options and strategies for tackling SARS-CoV-2 and COVID-19.

Notably, however, the current reality that COVID-19 infection rates and deaths in most African countries (and other countries considered underdeveloped), which were expected to buckle under the burden of COVID-19, are surprisingly much lower than predicted deserves to be investigated. Questions should be asked about what the people of those countries are doing to combat COVID-19 and whether or not, and to what extent, their massive use of traditional medicines plays a role in recording that relative success.

This review first explores natural products for use generally as antivirals, including their mechanisms of action. It then focusses on antiviral medicinal plants from the West African region, providing information about their identity, constituent compounds and their chemical structures, and the viral disease(s) they are used or reported to treat. It then discusses how these plants or herbal medicines containing them might be useful in the treatment of COVID-19 and similar coronavirus infections, based on their molecular mechanisms of action against other viruses, whether by direct antiviral effects or indirectly as anti-inflammatory and immunomodulatory agents. This work does provide detailed and contextualised understanding of the rationale and ramifications for the antiviral use of West African medicinal plants and how such existing knowledge repository and potential could be leveraged upon to investigate the plants for the treatment of COVID-19 or similar future infections, using an approach that integrates evidence-based herbal medicine into mainstream healthcare.

## Natural products and antiviral therapy

Evidence indicates that up to 80% of the population in developing countries use herbal medicines as the primary form of healthcare [9–11] due to several reasons, including relatively lower cost and perceived safety of traditional therapies compared with conventional medicines, unavailability or inaccessibility of conventional medical facilities and healthcare practitioners, and cultural and religious practices. Consequently, as COVID-19 emerges in those countries, it is not inconceivable that citizens will turn to herbal remedies for the prophylaxis, treatment, and symptomatic management of COVID-19. There are reports of the use of natural products and traditional medicines for such purposes.

Consistent with the fact that nature has influenced human health and well-being since ancient times, medicinal plants and other natural products have become integral components of health systems in developing countries [12, 13]. Modern drug discovery has also benefitted significantly from natural products [14–16].

The search for nature-derived or nature-inspired chemical leads that could be developed for the treatment of diverse diseases has also accelerated in recent years [17]. Scientists are increasingly exploring diverse natural sources: microbes, marine organisms and animals. In fact, there are numerous examples of antiviral drugs or drug candidates sourced from nature: Bevirimat (PA-457), an HIV maturation inhibitor and a semi-synthetic derivative of the ubiquitous betulinic acid (a triterpenoid) that is found in several species, including *Syzygium claviflorum* [18]; calanolide A, a pyranocoumarin non-nucleoside reverse transcriptase inhibitor (anti-HIV-1) from *Calophyllum lanigerum* [19, 20, 21]; ceglosivir, an alpha-glucosidase 1 inhibitor (for treating Hepatitis C Virus, HCV) that is a semi-synthetic derivative of castanospermine, an alkaloid from *Castanospermum australe* [22]; alisporivir, a cyclophilin-inhibiting anti-HCV drug, which is a non-immunosuppressive derivative of ciclosporin isolated from the fungus *Tolypocladium inflatum* [23] and has been reported to inhibit SARS-CoV-2 RNA production [24]; acyclovir (for treating herpes simplex virus infections, chickenpox and shingles) and zidovudine (anti-HIV), synthetic derivatives of arabinosyl nucleosides (nucleoside analogues) from *Tethya cripta* [25, 26] and cyanovirin-N, a protein with virucidal activity against several viruses (including HIV), isolated from the cyanobacterium, *Nostoc ellipsosporum* [27].

**Fig. 1:**
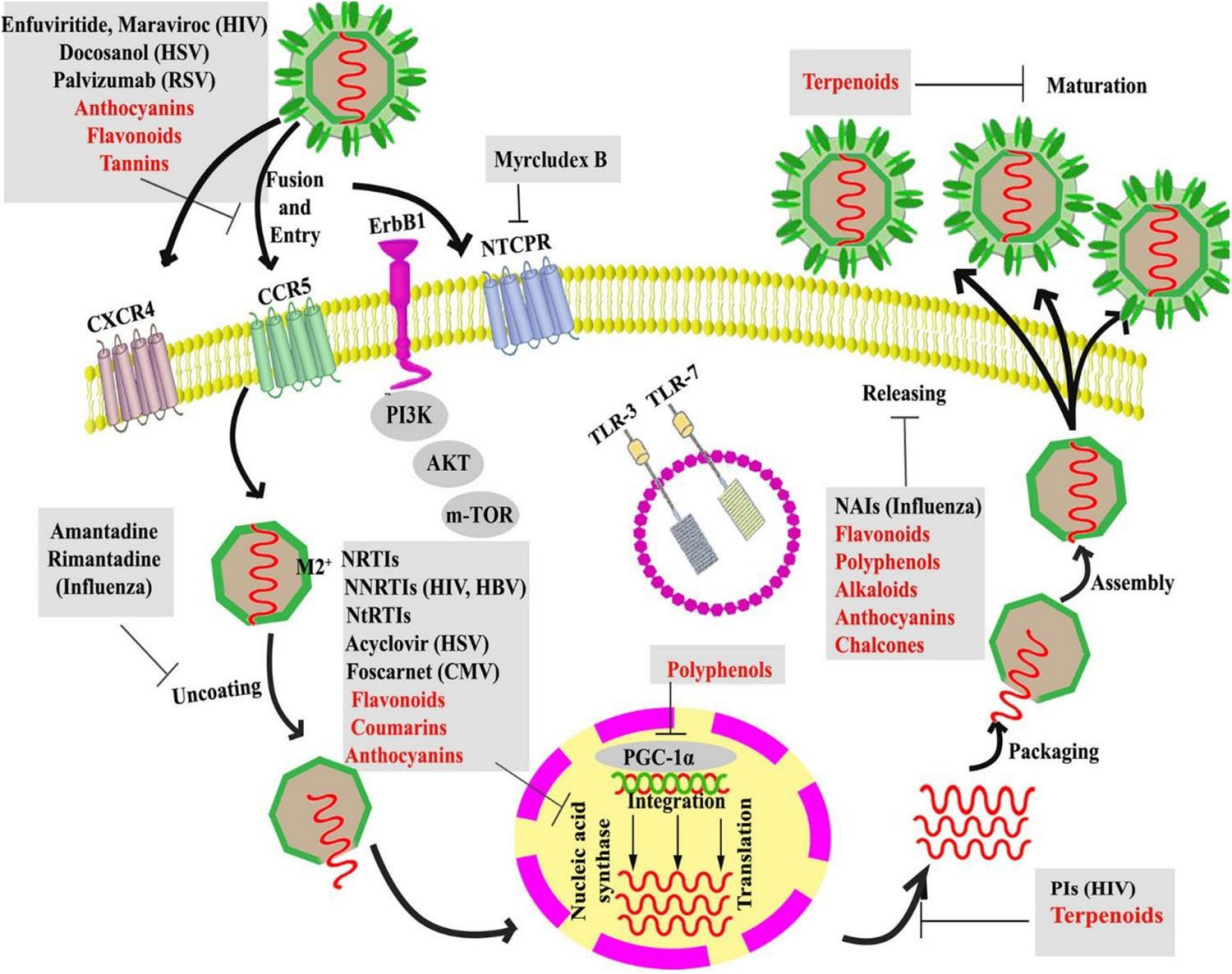
Mechanisms of antiviral action of various classes of natural compounds (indicated in red), with examples of some conventional antiviral therapeutics (for context and comparison), showing their extracellular and intracellular host- or virus-based drug targets. HIV, human immunodeficiency viruses; RSV, respiratory syncytial virus; HBV, hepatitis B virus; HSV-1/2, herpes simplex virus-1/2, NtRTIs, nucleotide reverse transcriptase inhibitors; NRTIs, nucleoside reverse transcriptase inhibitors; NNRTIs, non-nucleoside reverse transcriptase inhibitors; NAIs, neuraminidase inhibitors; PGC-1α, peroxisome proliferator-activated receptor gamma coactivator 1-alpha; CMV, cytomegalovirus; PI3K, Phosphatidylinositol-3-Kinase; TLR 3 or 7, toll-like receptor-3 or 7; AKT, Protein Kinase B; mTOR, mechanistic target of rapamycin; CCR5, C–C chemokine receptor type 5; CXCR4, C-X-C chemokine receptor type 4; ErbB1, epidermal growth factor receptor-1; NTCP Na + /taurocholate co-transporting polypeptide; PI, Protease Inhibitor. Figure reproduced with permission [35]

The majority of the antiviral herbs documented in literature have been found to contain active components such as flavones, alkaloids and polyphenols [28]. Flavonoids are said to constitute the largest source of antiviral agents in the entire plant kingdom [12]. For example, the flavone artogomezianone has been shown to possess anti-herpetic properties [29]; naringin has shown activity against HCV and HIV [30]; and quercetin reduced the infectivity and intracellular replication of Herpes Simplex Virus (HSV-1), Polio-virus type 1, Parainfluenza virus type 3 (Pf-3), and Respiratory Syncytial Virus (RSV) in cell culture monolayers [31]. Similarly, the alkaloid berberine, from Rhizoma Coptidis (RC), has been shown to prevent HSV penetration [32]; Farnsworth et al. [33] documented that nine of thirty-six alkaloids from *Catharanthus roseus* or *C. lanceus* were effective as antiviral agents, with pericalline being the most effective. Figure 1 shows the known or suggested mechanisms of antiviral action of flavonoids, polyphenols, terpenoids, coumarins, anthocyanins and chalcones, highlighting the various extracellular and intracellular drug targets, including host (entry) receptors and life cycle stages of the virus within the host. A recent review by Orhan and Senol Deniz [34] explored various articles from which they compiled the IC_50_/EC_50_ values for the anti-SARS-CoV activities of several flavonoids, some alkaloids, a few terpenes, diterpenes, saponins, diarylheptanoids and lectins, and a chalcone.

Despite the progress made in immunisation and antiviral drugs development, many viruses yet lack preventive vaccines and efficient and safe antiviral therapies. Thus, identifying novel antiviral drugs is of critical importance and natural products are an excellent source and may guide such discoveries [31, 36–38]. Interestingly, herbal remedies and natural products with antiviral activity have been mentioned in ethnobotanical surveys and reports of biological assays conducted in Africa. It should be emphasised that, beyond looking for chemical leads for the development of mono-component drugs, efforts, encouraged to be led by African nations, must also be directed towards deploying natural products known to have antiviral effects in developing standardised antiviral formulations, just as is now done with Traditional Chinese Medicine [39]. In this mini-review, we summarize available data on antiviral natural products, especially medicinal plants, focusing on those indigenous to, or found in, West Africa. We also highlight documented cases in the literature where these plants or constituents thereof have been shown to have positive effects specifically on coronaviruses.

## West African antiviral natural products

A review of medicinal plants in West Africa mentioned as part of ethnobotanical surveys for antiviral use within local populations and scientific investigations into possible antiviral properties showed that there are at least 124 species employed in West African traditional medicine (Table 1). These plants, whose leaves, roots, bark, flowers, latex and rhizomes form components of traditional antiviral remedies, are distributed across 50 plant families, exemplifying the recognised diversity of plants employed in traditional medicine systems [40–42]. The most prominent families were Amaryllidaceae, Anacardiaceae, Combretaceae, Compositae, Cucurbitaceae, Euphorbiaceae, Leguminosae, Malvaceae, Myrtaceae, Piperaceae, Rubiaceae, Rutaceae and Solanaceae. The Leguminosae and Compositae have been mentioned as part of the most species-rich medicinal plant families [43]. These plants (or the plant parts) are employed by the local population in the management of diseases, where viral infection is indicated, such as fevers, chickenpox, common cold, enteric conditions such as dysentery and diarrhoea, syphilis and other Sexually Transmitted Infections [44], measles, yellow fever, jaundice and hepatitis. From the literature review, one mushroom (*Hypoxylon fuscum*) *and* one lichen (*Ramalina farinacea*) were also reported to possess antiviral activities.

**Table 1:** West African Traditional Medicines with Suggested Antiviral Activity

**S/N**	**Traditional Medicine**	**Family**	**Common name**	** Part used**	**Local Indication**	**Investigated Antiviral Activity**	**References**
1	*Adansonia digitata *L.	Malvaceae	Monkey-bread tree	Bark, Root, Leaf	Intestinal and skin disorders, poliomyelitis asthma	NDV, HSV HCV, PV	[46–49]
2.	*Aframomum melegueta *K.Schum.	Zingiberaceae	Alligator pepper	Seed	Cholera, smallpox and chickenpox, measles	MV, YFV	[50–53]
3.	*Ageratum conyzoides *(L.) L	Compositae	Goat weed	Leaf, whole plant	Smallpox poliomyelitis, measles, yellow fever	EV 7, 19 HIV-1, HIV-2	[52, 54–56]
4.	*Allanblackia floribunba *Oliv.	Clusiaceae	Tallow tree	Leaf	Chickenpox, measles		[52]
5.	*Allium ascalonicum *L.	Amaryllidaceae	Shallot	Leaf, rhizome	Common cold Chickenpox		[52, 55]
6.	*A. sativum *L.	Amaryllidaceae	Garlic	Bulb	Poliomyelitis		[52]
7.	*Alstonia boonei* De Wild.	Apocynaceae	Cheese wood	Bark, Leaf	Yellow fever, jaundice		[52, 55]
8.	*Amaranthus viridis *L.	Amaranthaceae	Green amaranth	Leaf	Mumps	MV	[57, 58]
9.	*Anacardium occidentale *L.	Anacardiaceae	Cashew	Bark	Enteric conditions, worms, jaundice, measles, chickenpox, shingles	PV, AV, HSV 1, Equine HSV, BPV, CPV	[52, 59, 60]
10.	*Annickia chlorantha *(Oliv.) Setten & Maas	Annonaceae	African yellow wood	Bark	Fever, malaria	NDV	[61]
11.	*Anogeissus leiocarpa *(DC.) Guill. & Perr.	Combretaceae	African birch	Leaf	Fever, diarrhoea, dressings	PV, AV, HSV 1, Equine HSV	[59]
12.	*Argyreia nervosa* (Burm. f.) Bojer	Convolvulaceae	Elephant Creeper	Leaf	Chickenpox		[52]
13.	*Azadirachta indica* A. Juss.	Meliaceae	Neem tree	Leaf, bark	Fever, jaundice	DV, CV	[62, 63]
14.	*Bambusa vulgaris *Schrad.	Poaceae	Tropical bamboo	Leaf	Measles	MV	[50–52]
15.	*Bauhinia thonningii *Schum.	Leguminosae	Camel’s foot tree	Leaf	Diarrhoea, fever, influenza, cold, dysentery	PV, AV, HSV 1, Equine HSV, BPV and CPV	[59]
16.	*Boswellia dalzielii *Hutch.	Burseraceae	Nigerian Frankincense	Bark	Diarrhoea, fever, gastrointestinal disorders	PV, AV, HSV 1, Equine HSV, BPV, CPV	[59, 61]
17.	*Brachiaria ciliaris *Vanderyst	Poaceae	Buffalo grass	Leaf	Measles		[49]
18.	*Bryophyllum pinnatum* (Lam.) Oken	Crassulaceae	Life plant	Leaf	cold, pneumonia and respiratory tract infections, measles	EV 7, 19, HSV	[52, 54, 55]
19.	*Caesalpinia bonduc *(L.) Roxb.	Leguminosae	Warri tree	Leaf	Measles		[51]
20.	*Cajanus cajan *(L.) Millsp.	Leguminosae	Pigeon pea	Whole plant	Measles	MV	[52, 64, 65]
21.	*Capsicum annuum *L.	Solanaceae	Cayenne pepper	Seed	Measles		[51]
22.	*Carica papaya *L.	Caricaceae	Pawpaw	Leaf	Poliomyelitis, jaundice		[52, 64]
23.	*Cassia fistula *L.	Leguminosae	Golden shower	Seed	Common cold		[64, 66]
24.	*Ceratotheca sesamoides *Endl.	Pedaliaceae	False sesame	Leaf stem, root	Rhinitis, influenza, hepatitis, dysentery	MV	[57]
25.	*Chasmanthera dependens *Hochst.	Menispermaceae	Climbing plant	Leaf	Poliomyelitis		[52]
26.	*Citrullus colocynthis *(L.) Schrad.	Cucurbitaceae	Bitter cucumber	Seed	Measles		[51]
27.	*C. aurantiifolia *(Christm.) Swingle	Rutaceae	Lime	Fruit, leaf	Hepatitis measles, jaundice		[52, 64, 67]
28.	*C. paradisi *Macfad.	Rutaceae	Grapefruit	Leaf	Hepatitis		[64, 67]
29.	*Clausena anisata* (Willd.) Hook.f. ex Benth.	Rutaceae	Horsewood	Whole Plant	Whooping cough, syphilis, sore throat	HIV-1, HIV-2	[56]
30.	*Combretum indicum* (L.) DeFilipps	Combretaceae	Rangoon creeper	Leaf	Fever, Diarrhoea	FPV, NDV	[68]
31.	*C. mucronatum* Schumach. & Thonn.	Combretaceae		Leaf	Measles		[49]
32.	*Corchorus olitorius* L.	Malvaceae	Jute plant	Whole plant	Measles		[51, 52]
33.	*Crinum jagus* (J.Thomps.) Dandy	Amaryllidaceae	St. Christopher’s Lily	Bulb	Tuberculosis, epilepsy, asthma, infections	EV 7, 19	[54]
34.	*Cucumis metuliferus* E.Mey. ex Naudin	Cucurbitaceae	Horned melon	Fruit	Hepatitis, HIV/AIDS	NDV	[69]
35.	*Cymbopogon citratus* (DC.) Stapf	Poaceae	Lemongrass	Leaf	Jaundice, yellow fever		[52, 64]
36.	*Deinbollia pinnata* (Poir.) Schumach. & Thonn.	Sapindaceae	Indian beech	Seed	Measles		[51]
37.	*Detarium microcarpum* Guill. & Perr.	Leguminosae	Sweet detar	Bark	Dysentery, syphilis	HCV	[70]
38.	*D. senegalense* J.F.Gmel.	Leguminosae	Tallow tree	Leaf	Fever, dysentery, Boils	PV, AV, HSV 1, Equine HSV, BPV and CPV	[59]
39.	*Dichrostachys cinerea* (L.) Wight & Arn.	Leguminosae	Sickle bush	Leaf	Skin conditions, fever, diarrhoea measles, chickenpox, varicella	PV, AV, HSV 1, Equine HSV, BPV and CPV	[59]
40.	*Dioclea reflexa* Hook. f.	Leguminosae	Brown hamburger bean	Seed	Measles		[52]
41.	*Dioscorea cayennensis* Lam	Dioscoreaceae	Yellow yam	Leaf	Poliomyelitis		[49]
42.	*D. cayennensis subsp. rotundata* (Poir.) J.Miège	Dioscoreaceae	West African yam	Leaf	Measles		[51]
43.	*Diospyros barteri* Hiern	Ebenaceae		Leaf		PV Type 2	[71]
44.	*D. mespiliformis* Hochst. ex A.DC.	Ebenaceae	Jackalberry	Leaf, fruit, roots	Herpes, mumps, hepatitis	FPV, NDV	[68, 72]
45.	*D. monbuttensis* Gurke	Ebenaceae	Walking stick ebony	Seed	Herpes	PV Type 2	[71]
46.	*Ehretia cymosa* Thonn.	Boraginaceae		Leaf	Poliomyelitis, measles		[52]
47.	*Elaeis guineensis* Jacq.	Arecaceae	African oil palm	Oil	Herpes simplex, Measles		[51, 64]
48.	*Elytraria marginata* Vahl	Acanthaceae		Leaf	Measles		[51]
49.	*Emilia coccinea* (Sims) G.Don	Compositae	Tassel flower	Leaf	Mumps, herpes simplex, smallpox		[49]
50.	*Erigeron aegyptiacus* L.	Compositae		Leaf	Skin diseases, herpes, hepatitis	HSV, PV	[46, 47]
51.	*Eucalyptus camaldulensis* Dehnh.	Myrtaceae	Red river gum	Leaf	Fever, hepatitis, flu, rhinitis	PV type I, CV and EV 6	[73]
52.	*E. globulus* Labill.	Myrtaceae	Tasmanian blue gum	Leaf	Flu, fever, rhinitis	PV type I, CV and EV 6	[73]
53.	*Euphorbia Lateriflora Schumach*.	Euphorbiaceae	Crown of thorns	Leaf		MV	[57]
54.	*Ficus laurifolia* Lam.	Moraceae	Black fig	Root, bark	Tetanus convulsions	HSV	[46, 74]
55.	*Ficus polita* Vahl	Moraceae	Heart-leaved fig	Whole Plant	Hepatitis, fever	HIV-1, HIV-2	[56, 75]
56.	*Ficus thonningii* Blume	Moraceae	Common wild fig	Leaf	Jaundice, measles		[52]
57.	*Garcinia kola* Heckel	Clusiaceae	Bitter kola	Seed, root	Hepatitis, smallpox		[52, 64]
58.	*Gossypium arboreum* L.	Malvaceae	Tree cotton	Leaf	Hepatitis		[52]
59.	*G. barbadense* L.	Malvaceae	Egyptian cotton	Seed	Common cold		[49]
60.	*Guiera senegalensis* J.F.Gmel.	Combretaceae		Leaf	Enteric problems, Worms	PV, AV, HSV 1, Equine HSV	[59]
61.	*Hoslundia opposita* Vahl	Lamiaceae		Leaf	Measles, chickenpox, varicella		[76]
62.	*Hymenostegia afzelii *(Oliv.) Harms	Leguminosae		Fruit	Mumps		[64, 77]
63.	*H. fuscum* Pers. Fr.	Xylariaceae	Hazel woodwart	Whole mushroom		EV 7, 19	[78]
64.	*Hyptis pectinata* (L.) Poit.	Lamiaceae	Mint weed	Leaf	Poliomyelitis		[52]
65.	*Ipomoea asarifolia* (Desr.) Roem. & Schult.	Convolvulaceae	Ginger-leaf morning-glory	Leaf	Skin infections, abdominal cramps, diarrhoea	EV 7	[54]
66.	*Jatropha tanjorensis* J.L. Ellis & Saroja	Euphorbiaceae	Catholic vegetable	Leaf	Fever	HIV	[79]
67.	*Khaya ivorensis* A.Chev.	Meliaceae	African Mahogany	Bark	Jaundice		[52]
68.	*K. senegalensis* (Desv.) A.Juss.	Meliaceae	Khaya wood	Bark	Helminths	PV, AV, HSV 1, Equine HSV	[59]
69.	*Kigelia africana(Lam.) Benth.*	Bignoniaceae	Sausage tree	Bark	Poliomyelitis		[49]
70.	*Lactuca taraxacifolia* Schumach. & Thonn.	Compositae	African Lettuce	Leaf	Sores, measles, chickenpox, varicella	MV	[57, 80]
71.	*L. virosa* Habl.	Compositae	Wild lettuce	Bark	Poliomyelitis		[52]
72.	*Lagenaria breviflora* (Benth.) Roberty	Cucurbitaceae	Wild colocynth	Fruit, whole plant	Measles	NDV	[52, 66, 81]
73.	*Lannea humilis* (Oliv.) Engl.	Anacardiaceae		Bark	Diarrhoea, fever	PV, AV, HSV 1, Equine HSV	[59]
74.	*Lawsonia inermis* L.	Lythraceae	Henna tree	Leaf	Poliomyelitis, measles		[52]
75.	*Lippia multiflora* Moldenke	Verbenaceae	Bush tree	Leaf	Fever, ear and eye infections	EV 7, PV	[82]
76.	*Loranthus micranthus* Hook. f.	Loranthaceae	Green mistletoe	Leaf	Diarrhoea, diabetes, and microbial invasions	RSV	[83]
77.	*Macaranga barteri* Mull. Arg.	Euphorbiaceae	Macaranga plant	Leaf	Gonorrhoea, syphilis, skin infections	EV 7, 19	[54, 84]
78.	*Mangifera indica* L.	Anacardiaceae	Mango	Bark	Jaundice		[52]
79.	*Mimosa pigra* L.	Leguminosae	Giant sensitive plant	Leaf	Poliomyelitis		[52]
80.	*Mitracarpus hirtus* (L.) DC.	Rubiaceae	White eye	Leaf	Skin diseases	HSV, PV	[46, 47]
81.	*Momordica balsamina* L.	Cucurbitaceae	Balsam apple	Fruit Leaf	Measles, Yellow fever, skin disease	NDV, HIV	[51, 52, 85]
82.	*Mondia whitei* (Hook.f.) Skeels	Apocynaceae	White Ginger	Leaf	Malaria	EV 7, 19	[54]
83.	*Morinda lucida* Benth.	Rubiaceae	Brimstone tree	Roots	Yellow fever		[52]
84.	*M. oleifera* Lam.	Moringaceae	Moringa	Seed	Hepatitis	NDV	[85, 86]
85.	*Musa x paradisiaca* L.	Musaceae	Plantain	Leaf	Smallpox		[64]
86.	*Newbouldia laevis* (P.Beauv.) Seem.	Bignoniaceae	Boundary tree		Measles		[51]
87.	*Nicotiana tabacum* L.	Solanaceae	Tobacco	Leaf	Common cold, Poliomyelitis		[52, 64]
88.	*Olax subscorpioides* Oliv.	Olacaceae	Stink ant forest	Roots	Poliomyelitis		[52]
89.	*Palisota hirsuta* (Thunb.) K. Schum.	Commelinaceae		Leaf	Diarrhoea, skin disease	HSV, PV	[46, 47]
90.	*Parkia biglobosa* (Jacq.) G. Don	Leguminosae	African Locust Bean	Bark	Chickenpox, measles		[52]
91.	*Paullinia pinnata* L.	Sapindaceae	Supple jack	Whole Plant	Diarrhoea	HSV	[46, 47]
92.	*Peperomia pellucida* (L.) Kunth	Piperaceae	Pepper elder	Leaf, whole plant	Mumps, herpes simplex, measles		[51, 64, 77]
93.	*Persea americana* Mill.	Lauraceae	Avocado	Leaf	Poliomyelitis, hepatitis		[49]
94.	*Phyllanthus amarus* Schumach. & Thonn.	Phyllanthaceae	Sleeping plant	Leaf	Hepatitis, shingles	NDV	[87, 88]
95.	*Piper guineense* Schumach. & Thonn.	Piperaceae	West African Pepper	Seed	Measles,chickenpox		[52]^, [^ [51, 53]
96.	*Plumbago zeylanica* L.	Plumbaginaceae	Ceylon leadwort	Seed	Smallpox		[49]
97.	*Psidium guajava* L.	Myrtaceae	Common guava	Leaf, bark	Gastrointestinal disorders, jaundice	NDV	[52, [89]
98.	*Pycnanthus angolensis* (Welw.) Warb.	Myristicaceae	African nutmeg	Roots	Chickenpox		[52]
99.	*R. farinacea* (L.) Ach.	Ramalinaceae		Whole (Lichen)		HIV-1, Adenovirus, RSV	[90–92]
100.	*Raphia hookeri* G. Mann & H. Wendl.	Arecaceae	Ivory Coast raffia palm	Latex	Measles		[51]
101.	*Sarcocephalus latifolius* (Sm.) E. A. Bruce	Rubiaceae	African peach	Root	Jaundice, fever, diarrhoea, dysentery	RSV, NDV	[52, 93]
102.	*Securidaca longipedunculata* Fresen.	Polygalaceae	Violet tree	Seed	Smallpox		[49]
103.	*Senna occidentalis* (L.) Link	Leguminosae	Coffee weed	Leaf	Measles		[52, 51]
104.	*S. siamea* (Lam.) H. S. Irwin & Barneby	Leguminosae	Cassia tree	Bark		PV	[82]
105.	*S. singueana* (Delile) Lock	Leguminosae	Wild cassia	Leaf	Fever, worms	PV, AV, BPV, CPV	[59]
106.	*Sida acuta* Burm. f.	Malvaceae	Broom weed	Leaf	Yellow fever	HSV	[46, 64]
107.	*Solanum torvum* Sw.	Sapotaceae	Prickly solanum	Leaf	Yellow fever		[49]
108.	*Sphenocentrum jollyanum* Pierre	Menispermaceae	Morning seed	Leaf Root	Fever, hepatitis	PV Type 2	[94, 95]
109.	*Spondias mombin* L.	Anacardiaceae	Hog plum	Bark	Stomach ache, abdominal discomfort chickenpox, jaundice	EV 7	[52, 54]
110.	*Sterculia setigera* Delile	Malvaceae	Karaya gum tree	Bark	STIs, fever	PV, AV, HSV 1, Equine HSV, BPV and CPV	[59]
111.	*Symphonia globulifera* L.f	Clusiaceae	Boarwood	Root	Poliomyelitis		[52]
112.	*Terminalia ivorensis* A. Chev.	Combretaceae	Ivory Coast almond	Bark	Syphilis, burns, bruises, arthritis and haemorrhoids	EV 7	[54]
113.	*T. superba* Engl. & Diels	Combretaceae	Shingle wood	Bark	Yellow fever		[52]
114.	*Tetracera alnifolia* Willd.	Dilleniaceae	Ware vine	Leaf	Leprosy, cough	EV 7	[54]
115.	*T. potatoria* Afzel. ex G.Don	Dilleniaceae	Water tree	Bark	Jaundice		[52]
116.	*Uvaria chamae* P. Beauv.	Annonaceae	Finger root	Leaf, Bark	Fever, hepatitis	MV	[65, 96]
117.	*Vernonia amygdalina* Delile	Compositae	Bitter leaf	Leaf	Common cold, Measles, jaundice	VSV, PV, HSV	[51, 52, 77, 97]
118.	*Vitellaria paradoxa* C. F. Gaertn.	Sapotaceae	Shea tree	Fruits, Bark	Measles, Fever, dressing, Boils	PV, AV	[51, 59]
119.	*Vitex grandifolia *Gurke	Lamiaceae	Black plum	Leaf	Herpes simplex		[64, 77]
120.	*Xylopia aethiopica* (Dunal) A. Rich.	Annonaceae	Guinea pepper	Leaf, Bark, Fruit	Chickenpox, measles	MV	[52, 65]
121.	*Zea mays* L.	Poaceae	Maize	Flower	Chickenpox		[52]
122.	*Zephyranthes candida* (Lindl.) Herb.	Amaryllidaceae	White windflower			PV	[82]
123.	*Zingiber officinale* Roscoe	Zingiberaceae	Ginger	Rhizome	Yellow fever		[52]
124.	*Ziziphus mucronata* Willd.	Rhamnaceae	Buffalo thorn	Leaf	Enteric conditions	PV, AV	[59]

Table is an alphabetical list of plants employed as antivirals in traditional West African medicine (numbers 63 and 99 are not plants but a fungus and a lichen, respectively, but they were added for some context). The plant names, families, common names, part(s) employed as medicines, traditional indications, and viruses they are investigated for efficacy against are described

*AV* is Astrovirus, *BPV* is Bovine Parvovirus, *CPV* is Canine Parvovirus, *CV* is Coxsackie Virus, *DV* is Dengue Virus, *EV* is Echovirus, *FPV* is Fowlpox Virus, *HCV* is Hepatitis C Virus, *HIV* is Human immunodeficiency Virus, *HSV* is Herpes Simplex Virus, *MV* is Measles Virus, *NDV* is Newcastle Disease Virus, *PV* is Polio Virus, *RSV* is Respiratory Syncytial Virus, *VSV* is Vesicular Stomatitis Virus, *YFV* is Yellow Fever Virus

Over the years, the study of the therapeutic potentials of medicinal plants has not been consistently adequate, with only a small fraction of all flowering plant species in the world exhaustively studied for their potential pharmacological activity [9, 14, 45]. Consistent with this, in our review, only sixty-five (65/124; 52%) of the documented natural antiviral remedies have been scientifically evaluated for acclaimed therapeutic efficacies. Researchers have investigated the possible antiviral effects of these plants against RSV, Echoviruses, Measles Virus (Measles morbillivirus), HSV, HIV, Coxsackievirus and Dengue Virus. Others have also investigated the use of these plants against animal viruses such as Newcastle Disease Virus, Bovine and Canine Parvovirus, as well as Equine Herpesvirus.

**Fig. 2:**
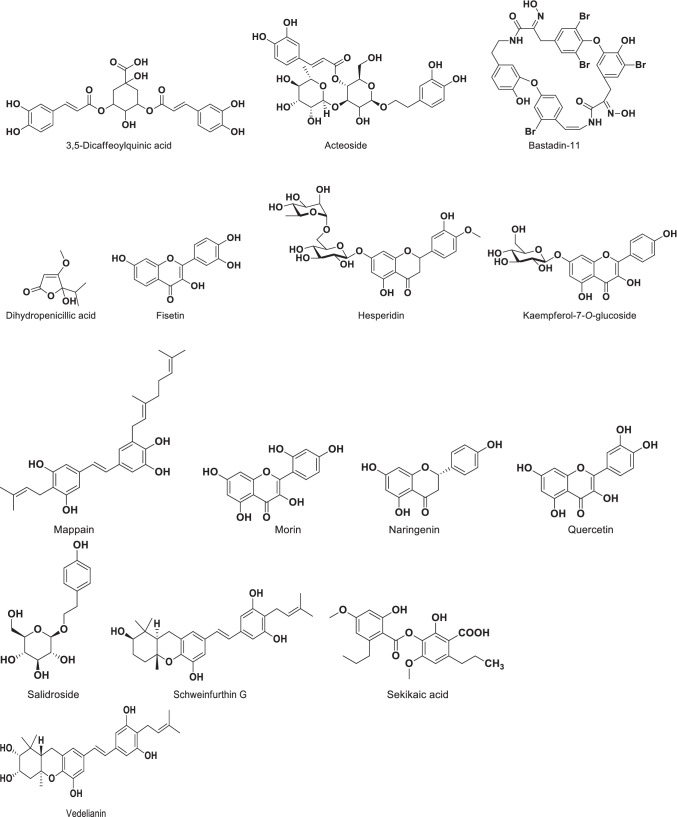
Chemical structures of compounds isolated from West African plants and reported to have antiviral activity

**Fig. 3:**
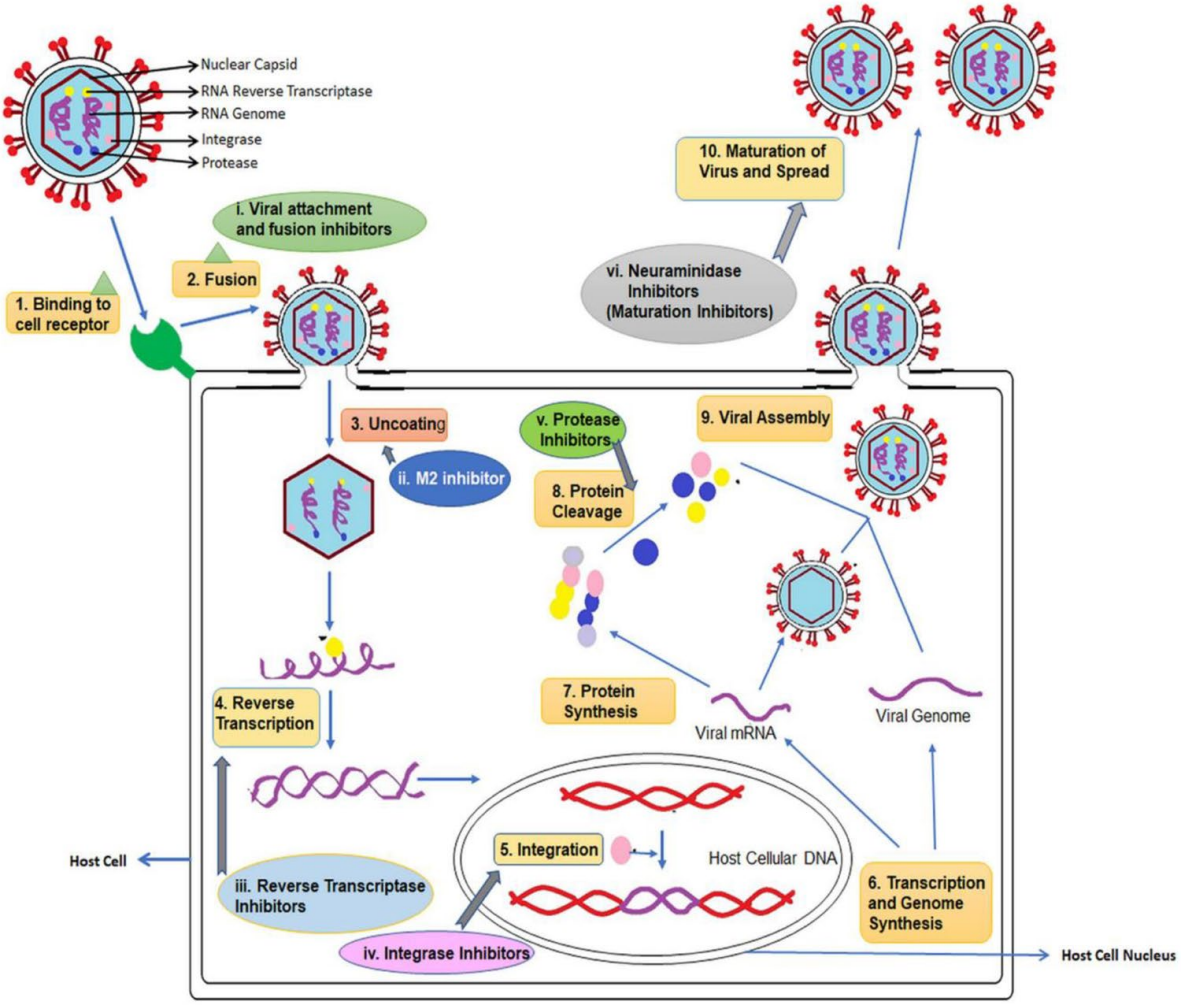
Antiviral drug targets that could mediate the antiviral effects of natural products. Figure reproduced with permission [98]

Of the 65 plants scientifically investigated, just four had their constituent phytochemicals potentially responsible for the observed activities isolated and identified, as shown in Table 2. The phytochemicals include the flavonoids quercetin, morin, fisetin, naringenin and hesperidin from *Citrus aurantifolia* and *C. paradisi*; alkaloids from *Cucumis metuliferus*; salidroside (2-(4-hydroxyphenyl)ethyl β-D-glucopyranoside) from *Loranthus micranthus*; flavonoids (3,5-dicaffeoylquinic acid, acteoside, kaempferol 7-*O*-glucoside, bastadin-11) and stilbenes (vedelianin, schweinfurthin G, mappain) from *Macaranga barteri*. In addition, dihydropenicillic acid was isolated as the active antiviral component of the mushroom *H. fuscum,* while sekikaic acid and other phenolic compounds were obtained from the lichen *R. farinacea.* The chemical structures of the compounds are shown in Fig. 2. We recommend that the drug targets mediating the antiviral activities of the remedies and isolated compounds should be investigated, using existing knowledge of the different potential antiviral drug targets as shown in Fig. 3.

**Table 2:** Compounds with antiviral activity which were isolated from antiviral West African Natural Products

**S/N**	**Natural Product**	**Constituent Antiviral Compounds**	**Antiviral Activity**		**References**
1	*H. fuscum*	Dihydropenicillic acid	Extract IC_50_ – EV7: 0.3811 µg/ml; EV19: 1.575 µg/ml		[78]
2	*M. barteri*	Flavonoids: 3,5-dicaffeoylquinic acid, acteoside, kaempferol-7- O-glucoside and bastadin-11 Stilbenes: vedelianin, schweinfurthin G and mappain	Mappain IC_50_ – EV7: 1.23 µM; EV19: 0.24 µM Vedelianin IC_50_ – EV7: 0.025 nM; EV19: 0.0036 nM Schweinfurthin G IC_50_ – EV7: 0.043 nM; EV19: 0.018 nM		[54, 84]
3	*C. aurantifolia*	Flavonoids: quercetin, motin, fisetin, naringenin, hesperidin			[64, 67]
	*C. paradisi*	Flavonoids: quercetin, motin, fisetin, naringenin, hesperidin			[64, 67]
4	*L. micranthus*	Salidroside (2-(4-hydroxyphenyl) ethyl-β-D-glucopyranoside)	Salidroside IC_50_—RSV: 10.3 ± 1.50 μg/ml		[83]
5	*R. farinacea*	Sekikaic acid (and other phenolic compounds)	Ethyl acetate-soluble fraction (ET4) IC_50_ – HSV-1: 6.09 μg/ml; RSV: 3.65 μg/ml; HIV-1: 0.33 μg/ml; HIV-1 RT 0.022 μg/ml Sekikaic acid IC_50_ Recombinant RSV: 5.69 µg/ml; RSV A2: 7.73 µg/ml		[91, 92]

Table lists antiviral compounds isolated from West African plants following investigations into their antiviral activity

*EV7* is Echovirus 7, *EV19* is Echovirus 19, *HIV* is Human Immunodeficiency Virus, *HIV RT* is Human Immunodeficiency Virus Reverse Transcriptase, *HSV* is Herpes Simplex Virus, *RSV* is Respiratory Syncytial Virus

## Potential of West African plants with antiviral activity as sources of drugs or herbal formulations to combat coronaviruses, including the current COVID-19 pandemic

While to date there are no direct ethnobotanical or other scientific reports from West Africa on the use of the plants listed in Table 1 against MERS-CoV, SARS-CoV or indeed SARS-CoV-2, there are numerous reports from elsewhere that suggest that natural products and traditional medicines may play a role in the fight against the current pandemic [99]. This work, therefore, highlights the potential of these plants to aid current and future drug discovery efforts aimed at identifying chemical leads for the development of anti-COVID-19 therapeutics, as well as the potential for developing the plants in the most easily acceptable forms as phytomedicines for the developing nations from where the plants originate. In this regard, it is important to note that, while the development of effective vaccines for the prevention of SARS-CoV-2 infection is considered a top priority in current thinking, the development of effective, anti-COVID-19 small-molecule drugs and phytomedicines should also continue to be prioritised, as any effective vaccines will have their limitations and contraindications, such that the need will always be there not only to prevent SARS-CoV-2 infection but also to treat those already infected or those who, for some reasons, are unable to access or be administered the vaccines. In a similar vein, with traditional medicines using natural products such as medicinal plants being part of the health care systems in some countries, the process of encouraging all hands globally to be on deck in tackling SARS-CoV-2/COVID-19 should include a clear recognition of the potential for such natural products to be part of the anti-COVID-19 armamentarium.

In some countries such as China and India in Asia and Mozambique in Africa, traditional medical remedies are officially recognised and integrated into the response to COVID-19. China’s response includes Traditional Chinese Medicine regimens such as the Lung Cleansing and Detoxifying Decoction (LCCD), which is widely used and approved by local authorities [99]. The decoction, amongst other things, contains *Dioscorea polystachya*, *Citrus aurantium* and Citrus peel. Both *Dioscorea* and *Citrus* species are mentioned in Table 1. An extract prepared from *Dioscorea* spp. patented in the USA (patent no. 20090041803) in 2008 was mentioned as potent against a host of viruses, including HSV-1, MV, RSV and SARS-CoV [28]. In a recent study, it was reported that many patients infected with COVID-19 in several African countries recovered from the infection using therapies made from herbal remedies which usually included garlic, ginger, lemon, turmeric, honey and neem (*A. indica)* leaves [100]. These reported therapeutic effects of those remedies are consistent with current evidence; for example, garlic is known to have antiviral properties [101]. The World Health Organization has approved a protocol for African herbal medicines to undergo clinical trials as potential treatments for COVID-19 and other epidemics and has also endorsed a charter and terms of reference to establish a data and safety monitoring board for the trials [102]. There is a recognition now that "the onset of COVID-19, like the Ebola outbreak in West Africa, has highlighted the need for strengthened health systems and accelerated research and development programmes, including on traditional medicines” [103].

**Fig. 4:**
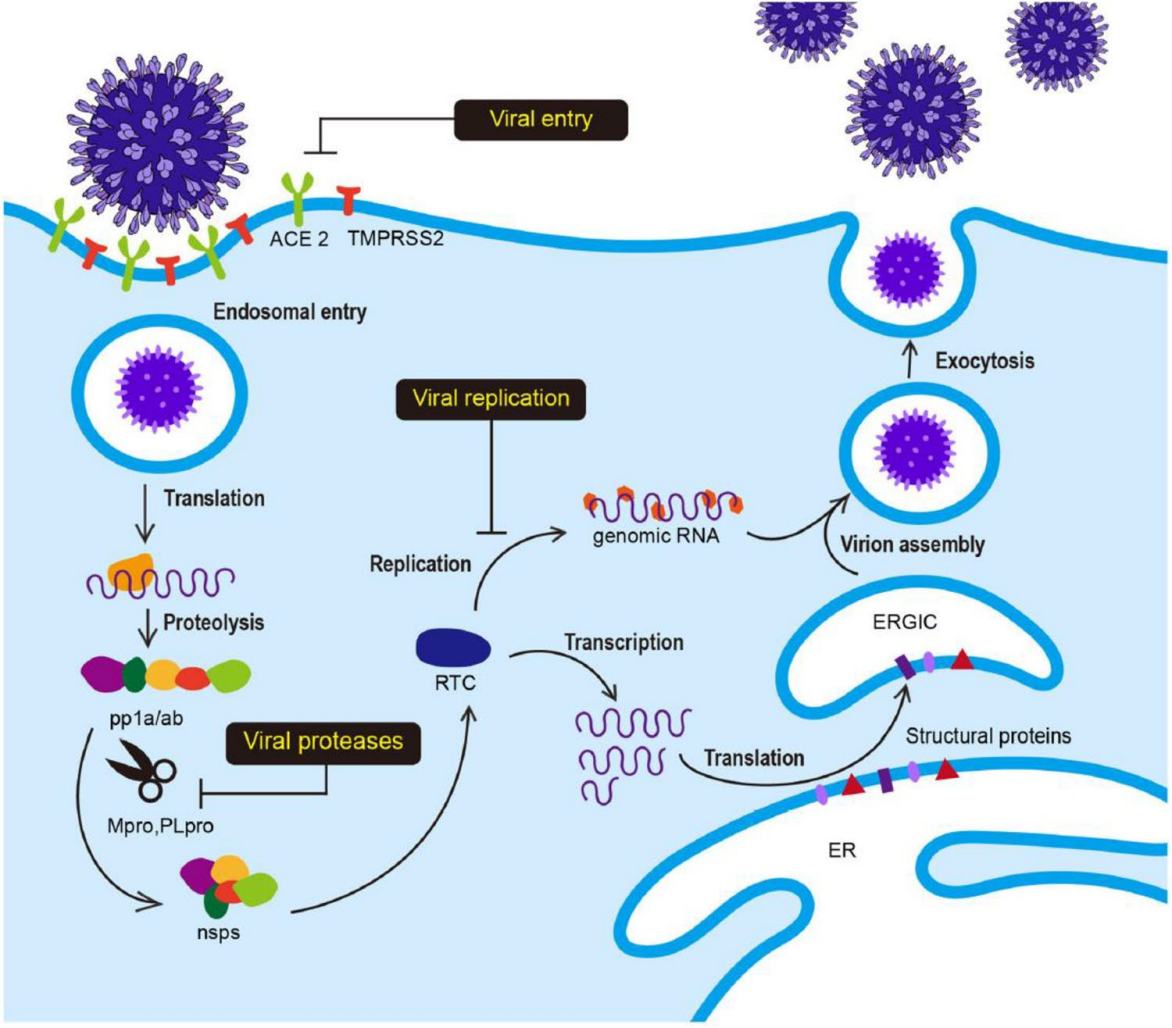
The life cycle of SARS-CoV-2 infection. The Angiotensin-Converting Enzyme 2 (ACE2) is a host entry receptor for viral entry, while Transmembrane Protease, Serine 2 (TMPRSS2) is a host serine protease that the virus uses to prime its spike (S) protein. The viral 3-chymotrypsin-like cysteine protease 3CL^pro^ (M^pro^) controls coronavirus replication. ER is Endoplasmic Reticulum, RTC is Replicase-Transcriptase Complex and ERGIC ER-Golgi Intermediate Compartment. Figure reproduced with permission [111]

**Fig. 5:**
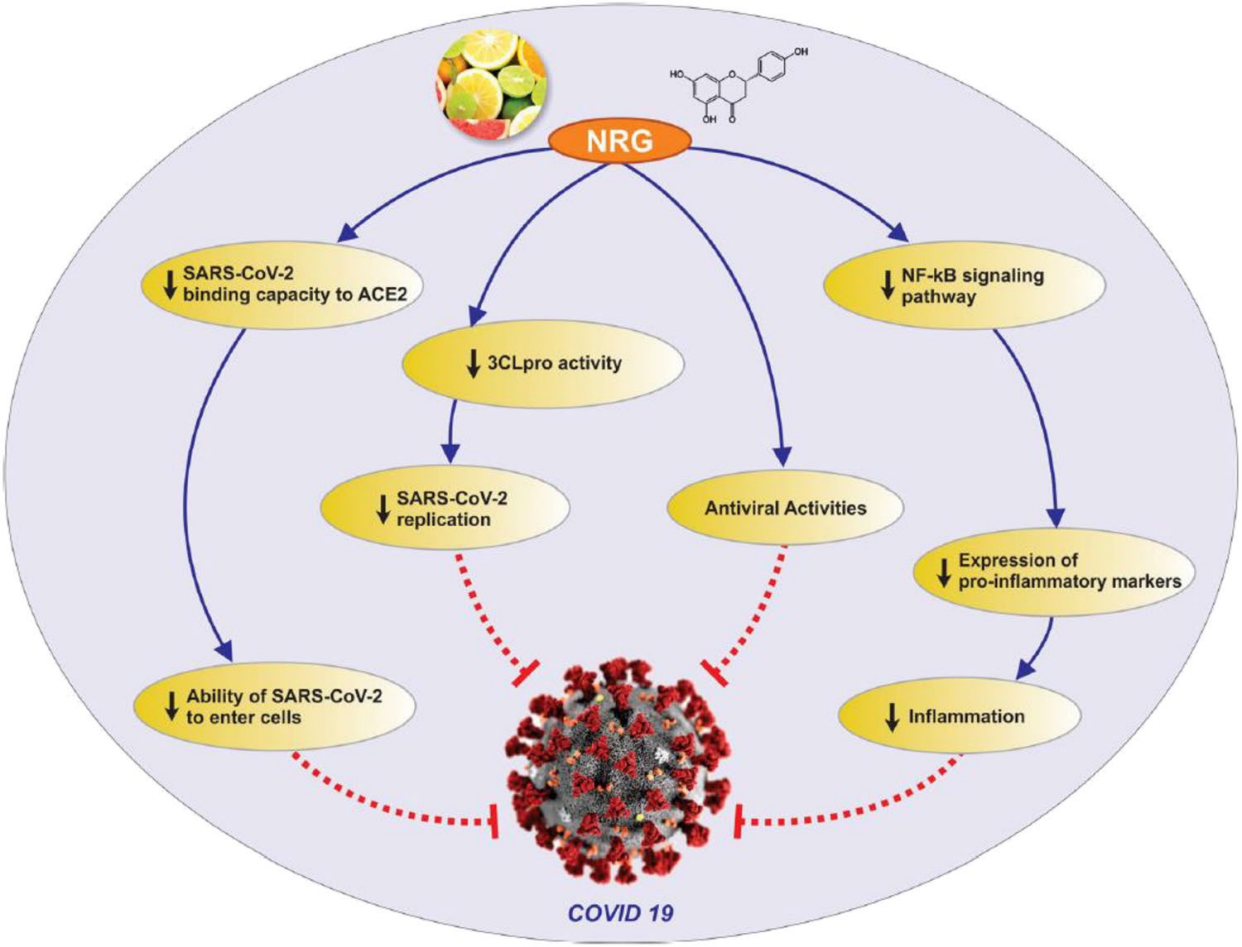
Antiviral and anti-inflammatory targets of the anti-COVID-19 activity of the natural compound naringenin. Naringenin targets the virus (SARS-CoV-2), as well as the inflammation associated with the infection (naringenin was one of the compounds isolated from some West African Citrus plants and reported to have antiviral activity). Figure reproduced with permission [112]

In the development of small-molecule therapeutics against SARS-CoV-2 (which causes COVID-19), many approaches have been identified, based on molecular targets linked to SARS-CoV-2 entry, replication and spike protein priming (see Fig. 4 for the life cycle of, and potential drug targets in, SARS-CoV-2). These approaches include binding to the viral 3-chymotrypsin-like cysteine protease 3CL^pro^ (M^pro^) enzyme that controls coronavirus replication and is essential for its life cycle [103]; inhibition of Angiotensin-Converting Enzyme 2 (ACE2), a host entry receptor for SARS-CoV-2; and inhibition of Transmembrane Protease, Serine 2 (TMPRSS2), a host serine protease that SARS-CoV-2 uses for its spike (S) protein priming [104] (Fig. 4). Interestingly, some natural compounds have been shown to possess efficacy against some of the targets [103]. Quercetin from Citrus fruits has been shown to have a high binding affinity for the SARS-CoV main proteinase (M^pro^ or 3CL^pro^) [105]. Hesperetin, an aglycone derivative of hesperidin and a naturally occurring flavanone-glycoside, the main flavonoid in lemons and sweet oranges, showed a concentration-dependent inhibitory effect on cleavage activity of 3CL^pro^ in cell-free (IC_50_ 60 µM) and cell-based (IC_50_ 8.3 µM) assays [106]. Hesperetin also showed significant ACE2 inhibition activity [107]. Both SARS-CoV and SARS-CoV-2 engage the receptor ACE2 for cell entry [104], thus suggesting possible anti-SARS-CoV-2 activity of hesperetin. Also, hesperetin, when used with chloroquine, had shown positive antiviral activity in vitro [108]. Other citrus flavonoids in lemon and orange peel, such as nobiletin, tangeretin and naringenin, have shown good affinities for SARS-CoV 3CL^pro^ and its receptors in molecular docking studies [105, 109, 110]. Naringenin was described in an earlier section as one of the compounds isolated from some West African Citrus plants and reported to have antiviral activity. Its mechanisms of anti-COVID-19 action, including directly targeting the virus as well as targeting the associated inflammation, are shown in Fig. 5.

In a separate assay to evaluate its inhibitory effect on ACE2, *C. aurantium* showed 100% activity, while *A. sativum* (Garlic) showed just above 70% activity [113]. *A. sativum* extract has been shown to possess activity against Infectious Bronchitis Virus (IBV), a coronavirus in poultry [114]. In a study that evaluated the activities of plant lectins against SARS-CoV using Vero and CrFK cells, *A. sativum* lectin was not active, but *A. porrum* (Leek) agglutinin was effective [115]. Alliin, a sulfoxide that is a natural constituent in fresh garlic, is a good inhibitor of SARS-CoV-2 M^pro^ as suggested by results of a molecular docking study [116].

Essential oil from Lemongrass (*C. citratus*) has been shown to exhibit anti-influenza activities [117]. Berberine, an alkaloid from *B. vulgaris*, has been found to significantly reduce RSV replication by reducing the synthesis of mRNA and viral proteins [118, 119]. Lactucopicrin-15-oxalate (from *L. virosa*, previously documented for antioxidant and antimalarial properties), biorobin (from *Ficus spp.*), and phyllaemblicin B (from *Phyllantus spp.*) were shown in in silico studies to have a high affinity for SARS-CoV-2 M^pro^, RNA-dependent RNA polymerase (RdRP) and human ACE2 [120].

Other studies have also reported the possible anti-coronavirus M^pro^ activities of rutin from *A. indica*, *T. chebula* and *O. basilicum*; amentoflavone from *M. indica* and *G. kola*; agathisflavone (a biflavonoid) from *A. occidentale*; rubusic acid from *S. nigrum*; chlorogenin from *S. torvum*; lupeol from *C. papaya* and *A. indica* and cyanin from *Z. officinale* [37, 121–123]. Nallusamy and colleagues [121] also showed that agathisflavone, corilagin (from *Terminalia* spp.) and cyanin have high binding affinities for the RdRP responsible for the replication of SARS-CoV-2.

*A. indica* has been widely considered to be of value against COVID-19 in Indian Traditional Medicine (Ayurvedic Medicine), where it is used to treat fever, cough, asthma and diarrhoea, which are associated symptoms of COVID-19. In an in vivo assay, it showed significant inhibitory activity against viral entry in mouse hepatitis virus (MHV) – a β-coronavirus—without adverse effects to the mice [124]. Nimocin, phytosterol, β-amyrin, nimbolin A are examples of phytoconstituents from *A. indica* with significant binding affinity and interaction with M protease of SARS-CoV-2 [125]. Another study showed that meliacinanhydride and other compounds such as nimocinol, isomeldenin, nimbolide and nimbin may be potential treatment options against COVID-19 [126]. Maurya et al. [127] also reported significant binding affinity of nimbin, piperine (from *P. guineense*), mangiferin (from *M. indica*) and berberine (from *Bambusa vulgaris*) for the spike glycoprotein of SARS-CoV-2, suggesting them as therapeutic or prophylactic options due to their inhibiting viral attachment.

*N*-acetyl glucosamine-specific agglutinins in *N. tabacum* showed positive results against SARS-CoV, with an effective concentration (EC_50_) of 1.7 ± 0.3 µg/ml and a cytotoxic concentration (CC_50_) > 100 µg/ml [115]. SARS-CoV has 23 putative *N*-glycosylation sites [128], and SARS-CoV-2 has been shown to have extensively glycosylated Spike protein on its surface [129]. Other studies have also recommended the use of *N. tabacum* as an oral vaccine (viral S or N antigen) [130, 131]. Of the 22 triterpenoids isolated from *E. neriifolia*, the frieldelane derivatives 3β-friedelanol, 3β-acetoxyfriedelane, friedelin and epitaraxerol showed significant anti-CoV activity in silico [132].

It is useful to remark that, concerning the development of phytomedicines, especially from medicinal food plants that have been used safely for hundreds of years, compounds that have been isolated from such plants and which show antiviral activity could be used as markers for quality assurance of the phytomedicines developed from them. Such products might not need to undergo the entire range of rigorous toxicity studies as are usually undertaken for isolated compounds, which when tested as single entities have been known to elicit toxicity not observed in the extract or the plant (containing them) when taken as such.

## Indirect anti-CoV activities of medicinal plants (anti-inflammatory and immunomodulatory effects)

Inflammation is now recognised as a critical mechanism in the pathophysiology of COVID-19. A sizeable number of COVID-19 patients develop cytokine storm, a severe hyper-immune response that leads to organ damage in some of those patients [133]. The use of some anti-inflammatory agents has recorded some degree of success in the management of the infection [134]. Some reports on the anti-CoV or anti-COVID-19 potentials of the plants detailed in Table 1 point to their significant immunomodulatory activities as a basis for such suggestions. Examples include:

- The hemicellulose fraction of *A. floribunda*, due to its significant antioxidant and immunomodulatory activities, especially its effect on Interferon-gamma (IFN-γ) production and Peripheral blood mononuclear cells (PBMC) [135].
- A garlic plus honey mixture may enhance the immune system due to the presence of sulphur-containing proteins and polyphenols [101, 136, 137].
- *M. indica* bark has shown possible immunomodulatory properties [138].
- *P. guineense*, *C. papaya*, *Z. officinale* and Citrus fruits all possess immunomodulatory properties [119].
- Ginger (*Z. officinale*), banana (*M. paradisiaca*) and *Solanum muricatum* are all suggested to develop the immunity of individuals against COVID-19 [139].
- *A. indica* possesses significant anti-inflammatory and potent immunostimulant activity [140].
- *C. fistula* is recommended in Unani Medicine for the preservation of health during epidemics because of its immunomodulatory and antioxidant properties [141].
- Naringin from citrus peel inhibits the expression of pro-inflammatory mediators COX-2, i-NOS, IL-1β and IL-6 in lipopolysaccharide (LPS)-induced RAW macrophages [142].
- Documented evidence shows that naringenin, the aglycone of naringin, might exert therapeutic effects against coronaviruses through the inhibition of 3CL^pro^ and reduction of ACE receptor activity. However, it might also exert a therapeutic effect against COVID-19 by attenuating inflammatory responses [143]. See Fig. 5.
- There have been calls for accelerated production of hesperidin-rich citrus pectin from citrus peels, as they possess immunomodulatory activity in addition to activity against 3CL^pro^ and ACE2 [144].
- *Dioscorea* plants have also shown immunomodulatory properties. Dioscorin, a tuber protein, possesses systemic and mucosal immunomodulatory activities [145]. It induces macrophage activation via stimulation of signalling molecules (ERK, JNK, NF-κB) and induction of pro-inflammatory cytokines (TNF-α, IL-1β and IL-6) [146, 147].
- Guava (*P. guajava*) leaf, mango (*M. indica*) stem bark and leaf, lemongrass (*C. citratus*) leaf, ginger (*Z. officinale*) rhizome, garlic (*A. sativum*) bulb and cinnamon (*Cinnamomum zeylanicum*) stem bark are immune-boosting herbs that are used in powdered form or as a decoction for oral administration [66].

## Conclusions and recommendations

The discovery and development of anti-coronavirus drugs, or specifically anti-COVID-19 drugs, including those from natural resources such as medicinal plants, will play a vital role in combatting the scourge of the current and future pandemics. Anecdotal knowledge is emerging of the successful use of certain medicinal food plants to manage symptoms of COVID-19. These natural resources and the knowledge of their therapeutic usefulness and promise abound in developing countries where, in contrast, the prohibitive cost of research on the development of synthetic drugs is generally unaffordable and technological facilities are lacking [148]. Considering this reality, alongside the added challenges posed by fragile and under-resourced health care management systems in many of those countries, the use of more affordable and more accessible herbal or other naturally-derived medicines to manage disease conditions, not least of which is the currently ravaging COVID-19, is undoubtedly an attractive alternative [149]. In line with this claim, the WHO also actively encourages these countries to develop and integrate traditional and alternative medicines into their health systems [150], as means to cope with their significant health care burden [151]. It is quite reassuring to note that, in many African countries, some phytomedicines to address serious disease conditions have now been well researched, packaged and produced, and some other phytomedicines are currently undergoing clinical trials, with yet some others in the pipeline. However, these research and development (R & D) efforts need to be further supported and expanded, including through substantial funding, both at the pre-clinical research level (high-throughput screening (phenotypic and target-based), phytochemical analysis, standardisation and quality control of herbs, dosage forms design, etc.), and clinical research level (involving clinical trials) [148].

This review briefly chronicles evidence demonstrating the rich diversity and potentials of medicinal plants in traditional medicine practice in West Africa for the treatment of viral infections. There is now an imperative to investigate, through coordinated approaches, these plants and their constituents for antiviral efficacy and safety. Collaborative, interdisciplinary studies involving scientists and indigenous people with authentic herbal medicine knowledge should be facilitated to promote antiviral drug discovery and identify herbal remedies and/or natural compounds that could be efficacious in preventing, treating, and managing symptoms of COVID-19 or other existing, emerging or future coronavirus diseases. Such cohesive research efforts ranging from the bench to the bedside could even furnish additional insights into disease mechanisms and therapeutics development beyond the antiviral domains of research and which encompass solutions to other areas of unmet clinical need.
